# Hepatoprotective effects and mechanisms of *Ixeris denticulate* water extract on liver cirrhosis in experimental rat

**DOI:** 10.1186/s12906-020-02957-w

**Published:** 2020-06-05

**Authors:** Yinhong Zhu, Changling Liu, Xiaobei Chen, Shengjia Lu, Jie Chen

**Affiliations:** Department of Infectious Diseases, Tongde Hospital of Zhejiang Province, No. 234 Gucui Road, Xihu District, Hangzhou, 321012 China

**Keywords:** Liver cirrhosis, *Ixeris denticulate*, Hepatoprotective, NF-κB p65, Bcl-2, Bax

## Abstract

**Background:**

To explore the protective effect and mechanisms of *Ixeris denticulate* water extract (IDWE) in the development of liver cirrhosis in experimental rat.

**Methods:**

Sixty rats were randomly divided into five groups: control group, model group and IDWE (2, 4 and 8 g/kg) treatment groups. Alanine transferase (ALT), aspartate transaminase (AST), albumin (ALB), tumor necrosis factor-alpha (TNF-*α*), Interleukin (IL)-6 and IL-8 in serum and superoxide dismutase (SOD), malondialdehyde (MDA) in liver tissue were evaluated, respectively. The liver index, liver morphology and liver histopathological analysis were detected as a supportive data. The liver protein expression of Bcl-2 and Bax were assessed by western blot, and NF-κB p65 protein expression was determined by immunohistochemistry analysis.

**Results:**

The result showed that a significantly decrease in the levels of serum AST, ALT and serum inflammatory factors TNF-*α*, IL-6 and IL-8 in IDWE-treated rats. The levels of serum ALB and SOD in liver tissue were markedly increased after IDWE treated, compared with model rats. Furthermore, IDWE-treated group also exhibited a down-regulated protein expression of NF-κB p65 and Bax, up-regulated Bcl-2 protein expression.

**Conclusions:**

IDWE could effectively alleviate the course of liver cirrhosis in rat model, which may be a potent hepatoprotective agent in clinical therapy in the future.

## Background

Liver cirrhosis is a common chronic liver disease in clinic, which caused by repeated actions of viral hepatitis, chronic alcoholism, autoimmune hepatitis and with a high fatality rate [1, 2]. The pathological characteristics of liver cirrhosis showed as follows: hepatocyte damage; liver showed a progressive, diffuse and fibrous pathological changes, which lead to destruction of normal structure and blood vessels of hepatic lobules, disorders of hepatic vascular structure and turbulence of blood circulation, formation of regenerative nodules and pseudolobules, proteogenesis disorders, hypoproteinemia with cirrhosis ascites in the late-stage, coagulation dysfunction and other clinical symptoms [3–5]. The above pathological characteristics will eventually lead to liver failure, portal hypertension, and multiple system involvement, and accompanied by various complications, such as ascites, hematemesis, jaundice and hepatic encephalopathy etc. [6, 7]. Significant abnormalities of liver function lead to liver functional failure, even the death of multiple organ failure [8]. According to statistics, the number of deaths caused by liver cirrhosis is about 500,000 ~ 700,000/year, accounting for 2.7% of all deaths in the world [2, 9, 10]. Therefore, liver cirrhosis has become a public health issue with a great concern.

However, there is still a lack of precise and effective therapeutic drugs in clinic. It is of great medical value to find new drugs that can effectively inhibit liver cirrhosis. At present, the treatment effect of single western medicine on liver cirrhosis is not ideal [11, 12]. *Ixeris denticulate* (also known as Baijiang, Kucai or Qumacao in China) is a dry whole plant of *Patrina scabiosaefolia* Fisch.ex Trev and *Patrinia villosa* Juss., which belongs to *Ixeris* of Valerianaceae. *Ixeris denticulate* was widely distributed in China, Japan and the former USSR, which mostly grow in wheat fields, vegetable fields or roadside [13, 14]. It is widely used as a food by people, since it contains a variety of amino acids, vitamins and minerals, and other beneficial ingredients. In addition, *Ixeris denticulate* is also a traditional Chinese medicine, which was widely used in the treatment of appendicitis, dysentery, tonsillitis, mumps and carbuncles, as well as in the treatment of hepatitis and liver cirrhosis [15, 16].

Chemical constitutes analysis showed that *Ixeris denticulate* mainly contains the activity constitutes of flavonoids and terpenoids [13]. In addition, the modern research found that some Chinese medicine, which with the activating blood circulation and removing blood stasis, have protective effects on hepatocytes, it can alleviate the damage of hepatocytes and inhibit the activation of HSC [17]. Liu et al. reported that the constitutes of *Ixeris denticulate* have an improving insulin resistance effect in 3 T3-L1 adipocytes in vitro [18]. Zou et al. found that *Ixeris denticulate* have a preventive effect for multipathogen induced pelvic inflammatory disease in rats [19]. While there also have some studies reported that *Ixeris denticulate* have the anti-inflammation effects in mice with ulcerative colitis [20], and anti-tumor effects in mice bearing U14 cervical cancer [21].

In this study, we aimed to explore and elucidate the hepatoprotective effect and the possible mechanisms of IDWE in carbon tetrachloride-induced liver cirrhosis in experimental animal in vivo. The study provided the experimental evidence for further clinical therapy of IDWE.

## Methods

### Materials and reagents

Alanine aminotransferase (ALT), aspartate aminotransferase (AST), albumin (ALB), superoxide dismutase (SOD) and malondialdehyde (MDA) reagents kit were obtained from Nanjing Jiancheng Bioengineering Institute (Nanjing, China). BCA protein assay kit was from Zhongshan Institute of Biotechnology (Beijing, China). Tumor necrosis factor-alpha (TNF-*α*), Interleukin (IL)-6 and IL-8 ELISA detection kit were purchased from R&D Company (USA). Rabbit anti-Bcl-2 and Bax antibody were purchased from cell signaling technology (CST, USA). Rabbit anti-NF-κB p65 was purchased from Abcam (USA). Horseradish peroxidase (HRP)-conjugated goat anti-mouse IgG, HRP-conjugated goat anti-rabbit IgG antibodies were provided by Proteintech Group, Inc. (Chicago, USA). CCl_4_ was obtained from Tianjin Shengtongtai Chemical Company (Tianjin, China). Peanut oil was obtained from Beijing Fangcao Pharmaceutical and Chemical Research and Development Company (Beijing, China).

### Preparation of IDWE extract

The crude dry *Ixeris denticulate* herb was purchased from Hangzhou Mintai Traditional Chinese Medicine Co., Ltd. (Hangzhou, China), and identified by Dr. Changling Liu (Department of Infectious Diseases, Tongde hospital of Zhejiang Province, Hangzhou, China). The voucher specimen was stored at herbarium of Tongde hospital of Zhejiang Province with the deposition number of ZYH-20171225.

Take proper mass of crude dry *Ixeris denticulate* herb to a ceramic pot (5 L), add 8-fold volume of distilled water (volume/weight), soak for 30 min, then boiling for 1 h, 45 min and 30 min, respectively. Combination of the three extracts, filtration with filter paper and condense to 150 ~ 200 mL. Then slowly add appropriate amount of 5% ethyl nipagin ester ethanol under rapid stirring. Finally, the gastric lavage liquid containing 2 g of crude drug per mL was prepared with distilled water at a constant volume of 900 mL for use.

### Animals

Male Sprague Dawley (SD) rats with body weight 150 ± 10 g were obtained from Experimental Animal Center of Zhejiang Province, China. Six rats were kept in one polyacrylic cages on a 12 h day/night cycle and quarantined for 1 week before the experiments. The rats were free access to food and water, housed in the standard controlled conditions with the temperature in 24 ± 1 °C, the humidity in 50 ± 5% and 12 h day/night cycle. The human care of the rats received was according to the National Institutes of Health Guidelines of China and related ethical regulations of Zhejiang Academy of Traditional Chinese Medicine. All the rats experiment were carried out at Zhejiang Academy of Traditional Chinese Medicine, and the rats were fasted for 12 h before sampling of material.

### Experimental design

When the rats were adapt to the environment for 1 week, seventy SD male rats were randomly divided into two groups. Group one as the control group were raised with normal feed (*n* = 12). Group two (*n* = 58) was used to establish the model, all the rats were intraperitoneal injection of 40% CCl_4_ oil solution (5 mL/kg), twice 1 week for 8 consecutive weeks. Then after modeling, Group two was divided into four groups: model, IDWE 2 g/kg, IDWE 4 g/kg and IDWE 8 g/kg groups (*n* = 16 for model, *n* = 14 for drug groups). Model and IDWE treatment groups were continue intraperitoneal injection of 40% CCl_4_ oil solution, and at the same time, orally administration with nature saline and the related doses of IDWE for model group and IDWE treated groups for 6 weeks, respectively.

After drug administration, all rats were sacrificed by cervical dislocation, blood samples and liver tissue of all rats were harvested for biomarkers assay, protein expression detection, liver histopathological analysis and the quantification of liver inflammation and fibrosis in rats. In addition, the survival ratio during the treatment period were calculated, the liver index was calculate as: Liver index % = liver weight/body weight × 100.

### Liver function biomarkers assay

The serum levels of ALT, AST and ALB were detected using commercial kits. Serum was collected from blood after the centrifugation at 3000 rpm for 10 min, 4 °C. The operation process of biomarker detection was according to the manufacturer’s instruction by a multifunctional analyzer Olympus AU600 (Tokyo, Japan). The enzyme activity of ALT and AST were calculated as U/L, and which observation absorbance were read at 505 nm. The serum levels of ALB was calculated as g/L, and the observation absorbance was read at 510 nm.

### Lipid peroxidation products and antioxidant enzyme activity detection

The contents of MDA in liver tissues was measured at 532 nm according to the protocol of commercial kit. The data of MDA was expressed as nmol/mg protein. Liver homogenate (10%, w/v) was prepared by homogenizing the liver tissue in 150 mM Tris-HCl buffered saline (pH 7.2) with a polytron homogenizer.

The enzyme activity of SOD in liver tissue was measured according to the commercial kit. The data are expressed as U/mg protein, and the observation absorbance of SOD was read at 550 nm.

### The serum levels of TNF-*α*, IL-6 and IL-8 assay

The serum levels of TNF-*α*, IL-6 and IL-8 were detected by commercial ELISA kit from R&D Company following the protocol provided by manufacture. The observation absorbance of TNF-*α*, IL-6 and IL-8 was read at 450 nm and the data are expressed as ng/mL for TNF-*α*, and pg/mL for IL-6 and IL-8.

### Liver morphology and histopathological observation

At the end of the experiment, the liver was obtained by caesarean section, observation and image the liver morphology. Then weight and cut about 1 cm × 1 cm × 1 cm liver specimen from the distance about 0.5 cm of the edge of the largest liver lobes. Liver specimens were fixed in 4% neutral formaldehyde buffer for overnight, and then embedded in paraffin, cut into 5 μm thickness for hematoxylin and eosin (H&E) staining. The H&E sections were examined and photographed under the Olympus BX-50 light microscope at 200× magnification.

In addition, the pathological stages of hepatic fibrosis were determined according to Metavir scoring system [22], F0 stage: normal liver tissue, no fibrosis; F1 stage: fibrosis extended to some portal areas; F2 stage: fibrosis extended to most portal areas; F3 stage: fibrosis extended to most portal areas, and portal fibrosis can be bridged opportunely; F4 stage: fibrosis extends to most portal areas, and there is characteristic bridging between portal fibrosis and central lobular fibrosis; F5 stage: there is characteristic bridging between portal fibrosis and central portal fibrosis, and there is opportunistic nodule formation.

### Immunohistochemistry analysis of hepatic NF-κB p65

The liver sections with 5 μm thickness were mounted on glass slides, then deparaffinized and incubated in 3% H_2_O_2_ for 10 min to quench endogenous peroxidase activity. The sections were stained with rabbit anti NF-κB p65 antibody at 4 °C overnight respectively, after blocking with normal goat serum for 20 min. Then incubation with HRP-conjugated goat anti-rabbit antibody at 37 °C, 30 min, respectively. The biding sites of the antibody were visualized by incubation with DAB-H_2_O_2_ at room temperature for 10 min. Images were taken at original magnification of 200× under Olympus BX-50 light microscope.

### Protein expression of Bcl-2 and Bax in liver tissue assay using Western blot

Protein levels of Bcl-2 and Bax in liver tissues were detected using Western blot. Liver tissue was washed with pre-cold phosphate buffer saline (PBS) buffer, then homogenized with the pre-cold tissue homogenizer in homogenate buffer (50 mmol/L, pH 7.5 Tis-HCl, 150 mmol/L NaCl, 1 mmol/L phenyl methyl sulfonyl fluoride, 1 mg/mL aprotinin, 4 mg/mL leupeptin) on ice bath. Then the homogenate centrifugated at 4 °C, 10000 rpm/min and collected the supernatant. Protein concentration was quantified by BCA kit. Take 50 μg protein samples to load at 12% SDS-PAGE electrophoresis gel for running, and then transferred from gel to PVDF membrane at 4 °C, 100 V for 1.5 h. First antibody dilution with the ratio of Bcl-2, 1:1000; Bax, 1:1000; beta-actin, 1:10000. After first and second antibody incubation, the membrane was exposure and imaged at Bio-Rad imager (Bio-Rad ChemiDoc MP, USA) with ECL (Enhanced chemiluminescent substrate for horseradish peroxidase (HRP), Thermo Fisher Scientific) reagent for 1 min.

### Statistical analysis

The data presented as mean ± SD. All statistical comparisons were made by One-way ANOVA test followed by Dunett’s t-test with GraphPad Prism 6.0 statistical software. *P* < 0.05 and *P* < 0.01 showed a statistically significant.

## Results

### General observation and liver index changes of all rats

**Fig. 1:**
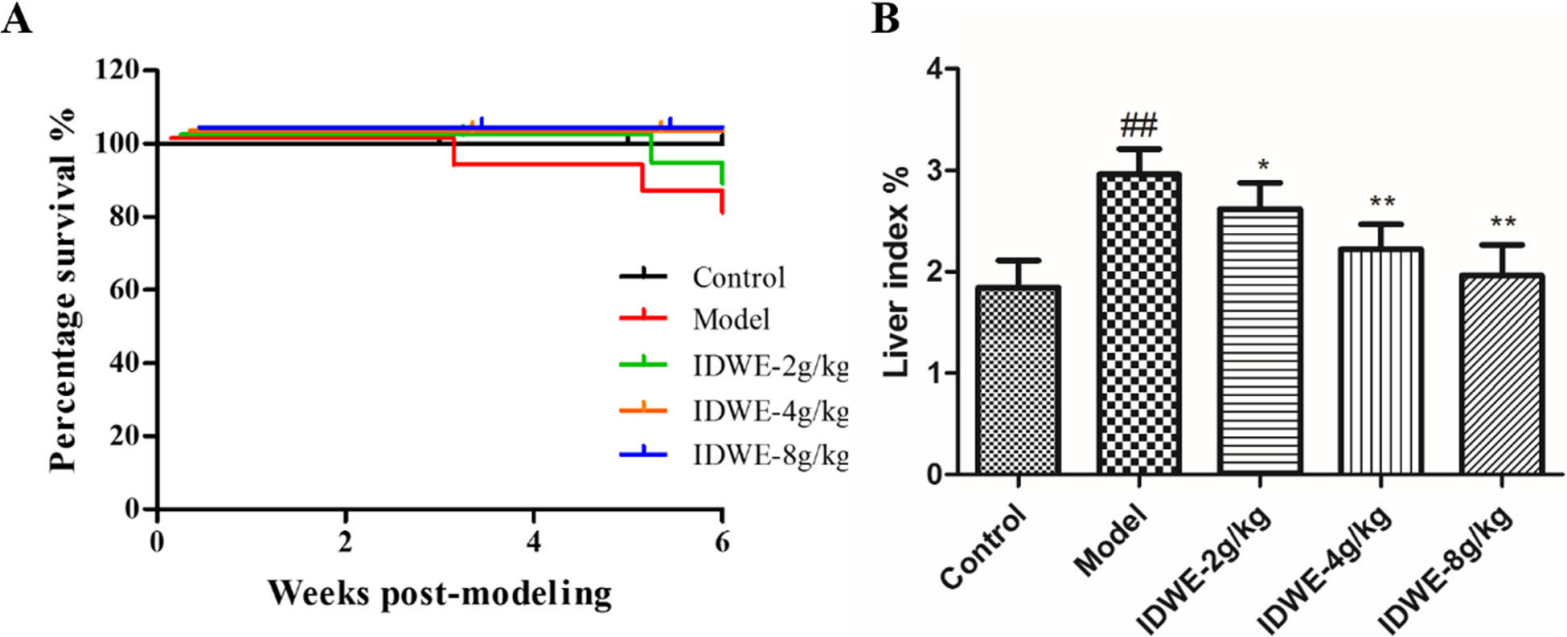
The survival curve and liver index analysis for all group rats (*n* = 12). A. Survival rate during the treatment period for all group rats, B. Liver index at the end of experiment. ^##^*P* < 0.01 vs control group, ^*^P < 0.05, ^**^P < 0.01 vs model group

Before modeling, the rats in each group had good mental state, glossy hair and normal drinking water. During the whole experiment, the rats in the control group had normal activity, sensitive reaction and glossy hair. From the 4th week, the rats in the model group were depressed, their hair was sparse, and their drinking water and food intake were reduced. At the end of the 6th week, rats developed ascites (abdominal puncture) in varying degrees. After administration, the mental state of rats in each drug dose group were improved, and at the end of administration, there was no obvious mental depression, no obvious abnormality in hair, drinking water and food intake in each drug group. In conclusion, during the modeling period, three rats died in the model group, two rats died in the low dose group, and no rats died in the other groups (Fig. 1a). The mainly reasons are liver necrosis, intestinal distention and malnutrition, skin ulcer and pulmonary infection failure.

In addition, the liver index in the model group was significantly increased, compared with the control group (*P* < 0.01, Fig. 1b). While after drug treatment, the liver index were markedly reduced, compared with the model group (*P* < 0.05, *P* < 0.01, Fig. 1b), especially in the high dose of drug treated group.

### The effect of IDWE on the levels of ALT, AST and ALB in serum

From Table 1, we can see that the levels of ALT and AST were significantly increased, while the levels of ALB was markedly decreased in model group, compared with control group (*P* < 0.01), which implied that CCl_4_-induced model has a significant effect on liver function and liver injury in rats. After drug treatment, IDWE significantly reduced the levels of ALT and AST, especially in the high dose of IDWE treatment group (*P* < 0.05, *P* < 0.01). Furthermore, IDWE also markedly increased the levels of ALB in serum, compared to the model group (*P* < 0.05, *P* < 0.01), which exhibited that IDWE has a protective effect in liver function and liver injury.

**Table 1:** The effect of IDWE on the levels of liver function marker

Group	ALT (U/L)	AST (U/L)	ALB (g/L)
Control	48.52 ± 7.64	79.56 ± 9.33	41.66 ± 4.18
Model	100.45 ± 13.77^##^	130.65 ± 16.38^##^	24.85 ± 4.59^##^
IDWE-2 g/kg	87.65 ± 10.32	115.71 ± 15.68	28.21 ± 3.67
IDWE-4 g/kg	65.31 ± 9.67^*^	99.56 ± 10.82^*^	36.44 ± 6.16^*^
IDWE-8 g/kg	49.47 ± 7.89^**^	81.03 ± 10.05^**^	38.93 ± 4.15^**^

Data are expressed as mean ± SD for each group (*n* = 12). ^##^*P* < 0.01 vs control group, ^*^P < 0.05, ^**^P < 0.01 vs model group. *IDWE Ixeris denticulate* water extract

### The effect of IDWE on the levels of TNF-*α*, IL-6 and IL-8 in serum

In Table 2, we detected the inflammation factors of TNF-*α*, IL-6 and IL-8 in serum of rats. The result showed that the levels of TNF-*α*, IL-6 and IL-8 significantly increased in model group, compared with the control group (*P* < 0.01), which have been caused organism inflammation. While after IDWE treated, the levels of the inflammation factors were significantly decreased, compared with the model group (*P* < 0.05, *P* < 0.01).

**Table 2:** The effect of IDWE on serum levels of TNF-α, IL-6 and IL-8

Group	TNF-α (ng/mL)	IL-6 (pg/mL)	IL-8 (pg/mL)
Control	3.79 ± 1.02	14.71 ± 2.11	0.22 ± 0.07
Model	9.93 ± 2.34^##^	39.12 ± 5.33^##^	0.99 ± 0.15^##^
IDWE-2 g/kg	9.27 ± 1.68	37.65 ± 6.13	0.79 ± 0.12
IDWE-4 g/kg	6.03 ± 1.45^*^	24.99 ± 3.16^*^	0.52 ± 0.08^*^
IDWE-8 g/kg	3.69 ± 1.32^**^	14.93 ± 1.97^**^	0.25 ± 0.05^**^

Data are expressed as mean ± SD for each group (*n* = 12). ^##^*P* < 0.01 vs control group, ^*^P < 0.05, ^**^P < 0.01 vs model group. *IDWE Ixeris denticulate* water extract

### The effect of IDWE on the contents of SOD and MDA in liver tissue

In liver tissue, we further detected the antioxidant enzyme activity of SOD and the contents of the lipid peroxidation products MDA. The data exhibited that, after modeling, the activity of SOD was significantly decreased, and the contents of MDA was significantly increased in model group, compared with control group (*P* < 0.01, Table 3). IDWE treated group markedly increased the activity of SOD, and significantly reduced the contents of MDA, compared with the model group (*P* < 0.05, *P* < 0.01), which implied that IDWE has the antioxidant effect.

**Table 3:** The effect of IDWE on the levels of SOD and MDA in liver tissue

Group	SOD (U/mg)	MDA (nmol/mg)
Control	228.35 ± 20.78	3.56 ± 1.01
Model	123.69 ± 15.61^##^	8.99 ± 1.25^##^
IDWE-2 g/kg	159.25 ± 11.37	7.42 ± 0.95
IDWE-4 g/kg	168.95 ± 10.42^*^	6.03 ± 1.11^*^
IDWE-8 g/kg	198.73 ± 17.85^**^	4.79 ± 1.20^**^

Data are expressed as mean ± SD for each group (*n* = 12). ^##^*P* < 0.01 vs control group, ^*^P < 0.05, ^**^P < 0.01 vs model group. *IDWE Ixeris denticulate* water extract

### Liver morphology observation

**Fig. 2:**
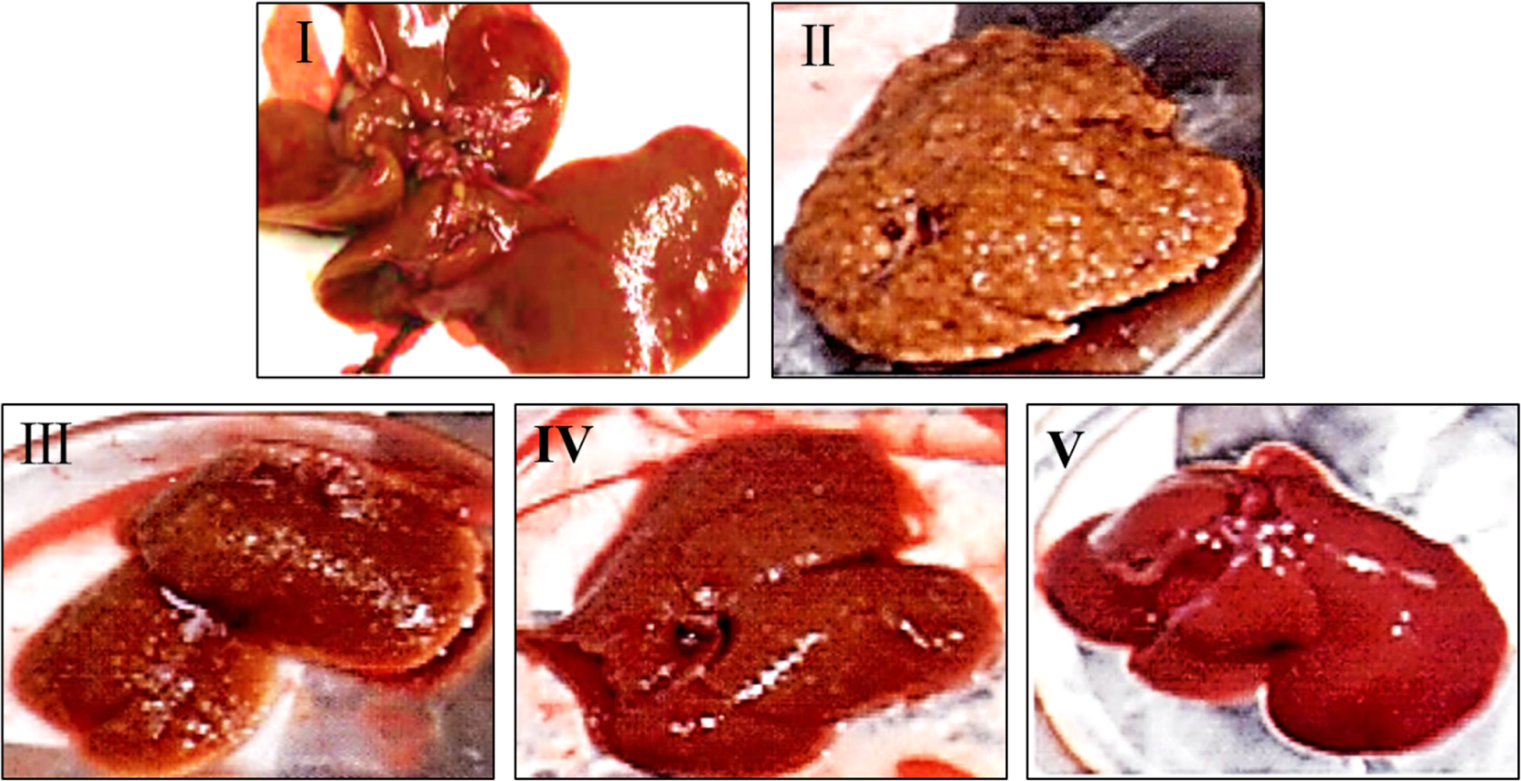
Liver morphology changes of rat in all groups. I, Control group; II, Model group; III, IDWE-2 g/kg; IV, IDWE-4 g/kg; V, IDWE-8 g/kg

At the end of the experiment, the liver morphology were observed by naked eyes, and captured the liver image. From Fig. 2, we can see that the liver of the rats in control group was ruddy and soft (Fig. 2i). The liver in model group was enlarged, dark and hard, and regenerative nodules could be seen (Fig. 2ii). While the volume, texture and color of liver tissues in IDWE treated groups were significantly improved (Fig. 2iii, iv, v).

### Histopathological analysis of liver tissue

**Fig. 3:**
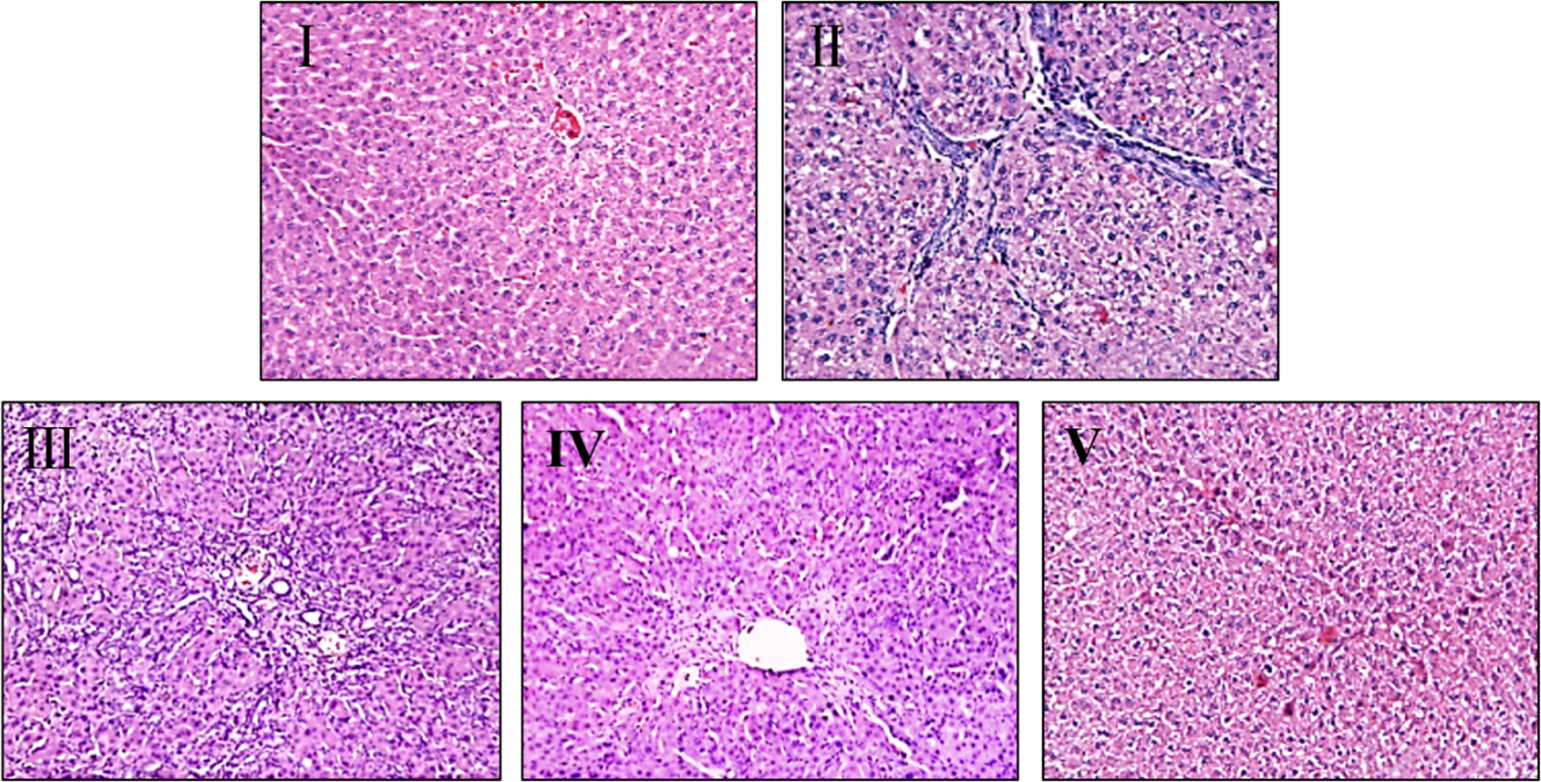
Histopathological changes of rat liver in all groups (200 ×). I, Control group; II, Model group; III, IDWE-2 g/kg; IV, IDWE-4 g/kg; V, IDWE-8 g/kg

The liver histopathological changes showed in Fig. 3. In control group, the structure of hepatic lobules was intact, the hepatocyte cords were arranged in a radial manner, and there was no degeneration and necrosis of hepatocytes, no expansion and congestion in the portal area of hepatic sinuses, and no infiltration of inflammatory cells (Fig. 3i). In model group, the structure of hepatic lobules was destroyed, hepatocytes showed fatty degeneration, focal necrosis, inflammatory cell infiltration, fibrous tissue proliferation and pseudolobule formation were observed in portal area and hepatic parenchyma (Fig. 3ii). Compared with the model group, the pathological changes of liver tissue in IDWE treatment groups were improved, especially in the high-dose group. The edema of liver cells was significantly reduced, the structure of hepatic cord was basically clear, slight fatty degeneration, a small amount of fibrous tissue proliferation in some portal areas, and inflammatory cells were significantly reduced (Fig. 3iii, iv, v). In addition, the histopathological stages of hepatic fibrosis were determined according to Metavir scoring system, the final result showed in Table 4.

**Table 4:** Grading of hepatic fatty lesions and inflammation of each group

Group	n	F0	F1	F2	F3	F4	F5
Control	12	12	0	0	0	0	0
Model	12	0	0	0	1	9	2
IDWE 2 g/kg	12	0	5	3	4	0	0
IDWE 4 g/kg	12	0	8	4	0	0	0
IDWE 8 g/kg	12	4	7	1	0	0	0

*IDWE Ixeris denticulate* water extract

### Effect of IDWE on the protein expression of NF-κB p65 in liver tissue by immunohistochemistry analysis

**Fig. 4:**
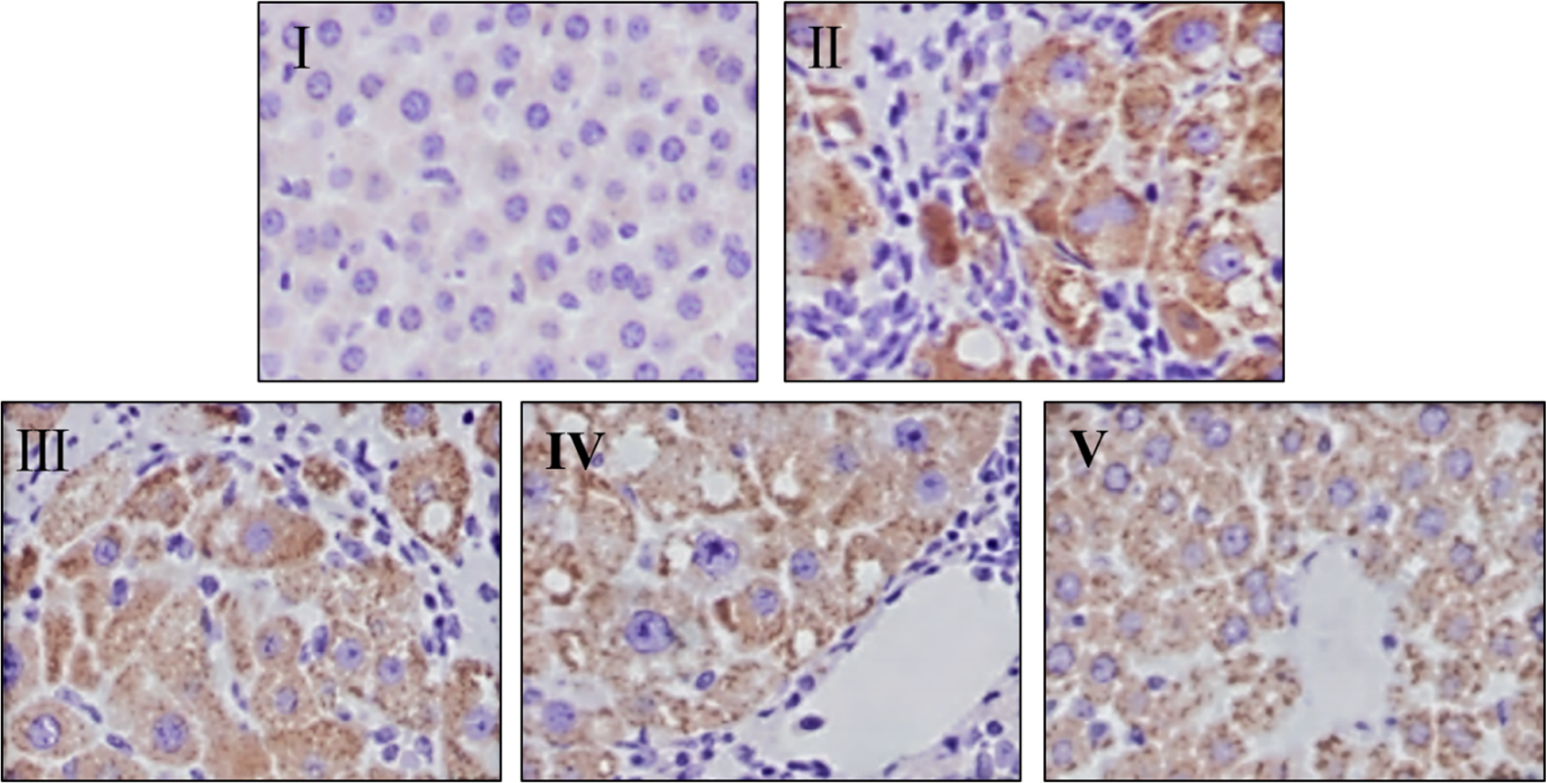
The protein expression of NF-κB p65 in liver tissue of all group rats (400 ×). I, Control group; II, Model group; III, IDWE-2 g/kg; IV, IDWE-4 g/kg; V, IDWE-8 g/kg

In addition, the protein expression of NF-κB p65 was detected through immunohistochemistry analysis (Fig. 4). As Fig. 4 shown the protein expression of NF-κB p65 markedly increased in model group, compared with control group (Fig. 4ii). Interestingly, IDWE treatment significantly decreased the protein expression of NF-κB p65, especially in the high dose of IDWE treatment group, compared with model group (Fig. 4iii, iv, v).

### Effect of IDWE on the protein expression of Bcl-2 and Bax by western blot analysis

**Fig. 5:**
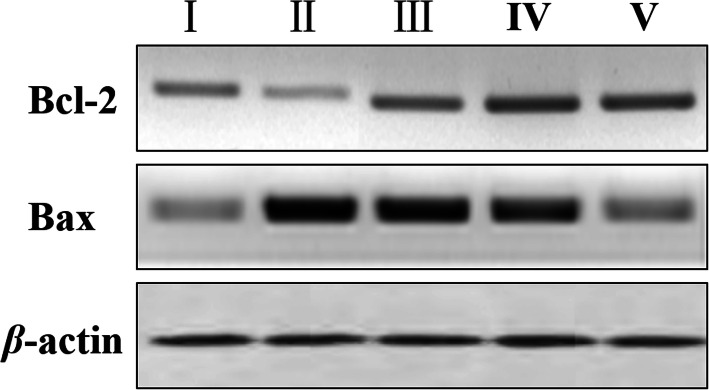
The protein expression of Bcl-2 and Bax in liver tissue of all group rats. I, Control group; II, Model group; III, IDWE-2 g/kg; IV, IDWE-4 g/kg; V, IDWE-8 g/kg

Based on the above results, we further detected the levels of apoptosis marker Bcl-2 and Bax by western blot analysis (Fig. 5). From the result, we could see that IDWE dramatically increased the protein expression of Bcl-2, and decreased the protein expression of Bax, compared with the model group (Fig. 5iii, iv, v), which reflected that IDWE could relieve the apoptosis of liver cell caused by liver injury.

## Discussion

Liver cirrhosis is a chronic liver disease with multiple clinical manifestations. It is displays diffuse hepatic nodules and depressions caused by various causes. Its clinical features are mainly ascites due to cirrhosis. The main pathological change of liver cirrhosis is liver fibrosis, which leads to a marked increase in the level of liver enzymes. It can also lead to the imbalance of synthesis and degradation of extracellular matrix and the changes of hemogram [23]. Carbon tetrachloride (CCl_4_), alcohol, immune, common bile duct obstruction and multi-factor combination method are the commonly used methods for preparing animal models of liver cirrhosis [24, 25]. Among them, CCl_4_ as a classical hepatotoxic substance is widely used in the preparation of liver cirrhosis model, which commonly used 40–60% CCl_4_-mixed oil solution twice a week for about 10 weeks [26]. In our study, 40% CCl_4_ oil solution was used for establishment of the liver cirrhosis model through repeated intraperitoneal injection in small dosage. The method need a short time and the progress of liver fibrosis is reliable, which is very suitable for the dynamic study of the occurrence and development of liver cirrhosis. Therefore, this model is mostly used in the development of new drugs for liver cirrhosis therapy [27, 28].

ALT and AST are abundant in hepatocytes. If hepatocytes are necrotic, they can be released into the blood circulation. The concentration level of ALT and AST can reflect the degree of hepatocyte damage to a certain extent [29]. The decrease of ALB is common in liver cirrhosis with ascites and other liver functions (such as acute hepatic necrosis, toxic hepatitis, etc.). Ascites is easy to occur when the ALB level below the normal level. MDA is the final product of lipid peroxidation, which can destroy cell membrane structure, leading to cell swelling, degeneration, necrosis and apoptosis. SOD is a superoxide free radical scavenging factor in the body, which can repair the damage of free radicals to hepatocytes. The content of MDA and SOD can reflect the degree of lipid peroxidation and indirectly reflect the degree of cell damage [29, 30].

The occurrence of complications such as ascites of cirrhosis in the later stage of liver cirrhosis is liable to form enterogenous endotoxemia, and the repeated enterogenous endotoxemia can activate hepatic mononuclear-macrophages to release TNF-*α*, IL-6, IL-8 and other inflammatory factors, leading to cascade reaction of inflammatory factors. It has been found that TNF-*α* is one of the main cytokines in the occurrence and development of hepatic inflammatory injury. It can promote the production and release of many other cytokines and eventually form a cytokine network to expand the inflammatory chain reaction [31, 32]. IL-6, produced by macrophages and monocytes, participates in the process of inflammatory damage and plays an important role in the local inflammatory response of the liver [33]. IL-8 is an important leukocyte chemokine, which can cause inflammation by promoting the aggregation of inflammatory cells into the liver, and then lead to the damage of hepatocytes [34]. However, many molecules involved in the early stage of immune response and inflammatory response are regulated by NF-κB, including TNF-*α*, IL-1*β*, IL-2, IL-6, IL-8, IL-12, iNOS and COX2 etc. As an early transcription factor, the activation of NF-*κ*B does not need to be regulated by newly translated proteins. Therefore, it is possible to respond to the stimulation of harmful cells at the first time [35, 36].

Hepatocyte apoptosis is one of the most important causes of liver diseases, and plays an important role in normal liver development and the occurrence of various liver diseases [37]. When the liver is stimulated by injury factors, such as alcohol, virus infection, the death process of hepatocytes may be activated, which may lead to programmed death (apoptosis) of hepatocytes, and produce a large number of apoptotic bodies. The main phagocytes in the liver engulf apoptotic bodies, and a large number of Kupffer cells are engulfed after they are engulfed. Pro-inflammatory factors and apoptotic receptors can further promote the apoptosis of hepatocytes and trigger inflammation, leading to the formation of liver fibrosis [38]. Therefore, it is believed that pathological apoptosis of hepatocytes is closely related to the occurrence of hepatic fibrosis. Pathological hepatocyte apoptosis initiates and promotes the occurrence of hepatic fibrosis.

Bcl-2 family is the most widely studied apoptosis-related genes. Its members include two types: one is apoptosis-promoting genes, including Bax, Bad, Bak and so on. The other is the apoptosis suppressor gene represented by Bcl-2. Bcl-2 protein mainly distributes in the outer membrane of mitochondria, the inner surface of cell membrane, endoplasmic reticulum and nuclear membrane [39]. Bax is more widely expressed in hepatocytes, vascular smooth muscle cells and so on. It was found that when there were more Bcl-2 in cells, the heterodimers of Bcl-2 and Bax increased and the apoptotic tendency decreased. When there were more Bax in cells, the homologous dimers formed by Bax itself dominated, and the apoptotic rate was prone to occur. The ratio of Bcl-2/Bax was important to determine whether the cells entered the apoptotic state or not [40].

In the study, IDWE can reduce the elevate ALT and AST, increase the decreased ALB induced by CCl_4_. It can also reduce the lipid peroxidation products MDA contents, enhance the activity of antioxidant enzyme SOD. In addition, IDWE reduced the levels of TNF-*α*, IL-6 and IL-8, thus reducing the infiltration of hepatic inflammatory cells and the protein expression of NF-κB p65. Furthermore, the results showed that Bcl-2 and Bax were involved in the process of liver cirrhosis. IDWE can inhibit or reduce the expression of apoptotic protein Bax and increase the expression of anti-apoptotic protein Bcl-2, thereby inhibiting and reducing hepatocyte apoptosis. IDWE can reduce transaminase and improve liver function, at the same time, it can also reduce the apoptosis of hepatocytes and protect liver tissue.

In addition, the lack of detailed component analysis of the IDWE in this study is a limitation to this study. Furthermore, the isolation and identification of the chemical constitutes of IDWE is underway, the chemical constitutes and the hepatoprotective constitutes analysis in cellular levels in vitro will be reported in due course.

## Conclusion

In summary, IDWE exhibited a significant effect against CCl_4_ induced liver cirrhosis, which may be probably due to the antioxidant effect, relieve the inflammation reaction, down-regulated protein expression of NF-κB p65 and Bax, up-regulated Bcl-2 protein expression. The result implied that IDWE may be a potent hepatoprotective agent in clinical therapy in the future.

## Data Availability

The data used and/or analyzed during this study can be obtained from the corresponding author with a reasonable request.

## Abbreviations

IDWE: *Ixeris denticulate* water extract
ALT: Alanine transferase
AST: Aspartate transaminase
ALB: Albumin
TNF-*α*: Tumor necrosis factor-alpha
IL-6: Interleukin-6
SOD: Superoxide dismutase
MDA: Malondialdehyde
SD: Sprague Dawley
CCl_4_: Carbon tetrachloride
BW: Body weight
